# Enhanced Droplet Control by Transition Boiling

**DOI:** 10.1038/srep00720

**Published:** 2012-10-10

**Authors:** Alex Grounds, Richard Still, Kei Takashina

**Affiliations:** 1 Department of Physics, University of Bath, Bath BA2 7AY, United Kingdom

## Abstract

A droplet of water on a heated surface can levitate over a film of gas produced by its own evaporation in the Leidenfrost effect. When the surface is prepared with ratchet-like saw-teeth topography, these droplets can self-propel and can even climb uphill. However, the extent to which the droplets can be controlled is limited by the physics of the Leidenfrost effect. Here, we show that transition boiling can be induced even at very high surface temperatures and provide additional control over the droplets. Ratchets with acute protrusions enable droplets to climb steeper inclines while ratchets with sub-structures enable their direction of motion to be controlled by varying the temperature of the surface. The droplets' departure from the Leidenfrost regime is assessed by analysing the sound produced by their boiling. We anticipate these techniques will enable the development of more sophisticated methods for controlling small droplets and heat transfer.

From nanofluidics, lab-on-a-chip applications in microfluidics to ink-jet printing, the physics and technology of controlling small quantities of liquid continues to attract wide interest^1^,^2^,^3^,^4^,^5^,^6^,^7^,^8^,^9^,^10^,^11^. It has recently been discovered that droplets of liquid on a hot surface can propel themselves and even climb up hill if the surface is textured with a periodic saw-teeth-like ratchet topography^1^. While this offers interesting new possibilities for manipulating droplets, the extent to which the droplets can be controlled is determined by the physics of the Leidenfrost effect^12^,^13^,^14^ and the mechanisms underlying the propulsion^1^,^3^,^5^,^15^.

A droplet in the Leidenfrost regime is suspended on a cushion of gas evaporating from its surface through film-boiling. On a ratcheted surface, the surface structure provides an asymmetry for the gas to flow asymmetrically, leading to a net lateral force on the droplet allowing it to be propelled. This net force, however, is limited by a number of factors. On one hand, increasing the temperature of the surface beyond the Leidenfrost point increases the heat transferred to the droplet which increases the gas flow from the droplet. However, this enhances the levitation of the droplet which in turn results in a loss of traction so that increased surface temperature can lead to very little increase in acceleration^1^, or even a reduction depending on the detailed conditions. On the other hand, reducing the temperature of the surface into the transition boiling regime dramatically increases the heat transfer rate and the acceleration is strongly enhanced^1^, although at too low a temperature, the droplet makes too much contact with the surface leading to the loss of propulsion.

It is well known, however, that the Leidenfrost point is strongly affected by the surface roughness^16^,^17^. It is therefore natural to expect that ratcheted surfaces have effective Leidenfrost temperatures that differ from a flat surface, and that this temperature also depends upon the ratchet design. It follows further that there should be a temperature-scale associated with a size-scale of the features on the surface, in turn leading to a possibility of controlling how acceleration of the droplets vary with temperature by embedding different levels of structure on the solid surface. Here, we demonstrate that ratchets with acute protrusions significantly raise the Leidenfrost temperature and that they enable droplets to climb steeper inclines. Furthermore, we show that ratchets with sub-structures enable the direction of motion to be tuned and varied by controlling the temperature of the surface.

## Results

### Droplets climbing uphill

**Figure 1:**
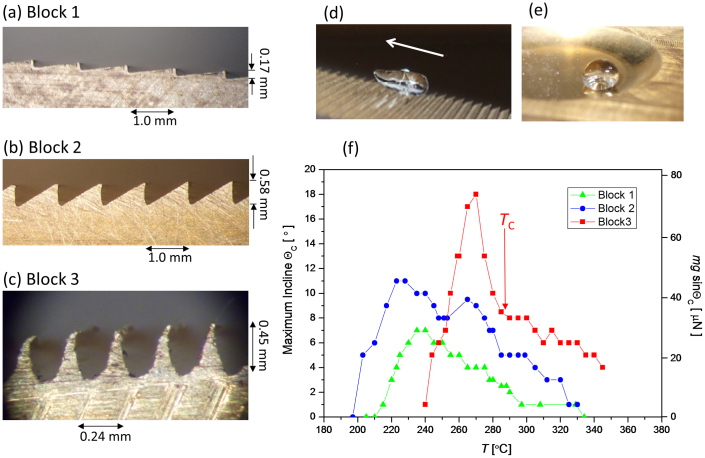
Ratchets and droplets. (a), (b) and (c) show photographs of Blocks 1, 2 and 3 respectively. Droplets travel from right to left. (d) A droplet climbing Block 2. The arrow indicates the direction of motion. (e) A droplet in a flat bowl. (f) Maximum incline the droplets were able to climb for the three blocks. The axis on the right hand side shows the corresponding gravitational force in the backwards direction. *T*_C_ marks the temperature at which the temperature dependence suddenly changes as described in the body text.

On a rough surface, the Leidenfrost effect is achieved when the droplet can levitate itself to a sufficient height that there is no direct contact between the droplet and the most protruding feature of the surface. Thus, surfaces with sharper and more protrusive ratchet teeth can be expected to raise the Leidenfrost temperature, and in turn, enable droplets to be propelled with more power and climb steeper inclines. We present data from three ratchets with different sharpness of teeth as shown in Figs. 1 (a), (b) and (c). These were prepared on separate blocks of brass: Block 1 had a pitch of 1 mm where the sloping part of the ratchet had an incline of 10°, Block 2 also had 1 mm pitch but with sloping parts of teeth at 30° while Block 3 had acute but asymmetric teeth and a shorter pitch of 0.24 mm. Details of their fabrication are detailed in the Methods section. The blocks were heated on a hotplate and droplets of water with a spherical diameter of 3.6 mm were dropped on them. The incline of the block with respect to the horizontal, *θ*, was increased until the droplets were no longer able to climb (*θ*_C_) and this angle was recorded [Figure 1 (f)].

At the highest temperatures, all three blocks show a similar trend whereby *θ*_C_ decreases with increased temperature. i.e. increasing the temperature reduces the ability of the droplets to climb. This is characteristic of droplets in the Leidenfrost limit where at higher temperatures; the droplets are forced further away from the surface. With temperature reduced, *θ*_C_ peaks before dropping down to zero. As expected, Block 3 with the sharpest teeth shows the largest peak value of *θ*_C_ and furthermore, the peak lies at a substantially higher temperature compared to the other two blocks. We interpret this to be due to the differences between the Leidenfrost temperatures *T*_L_ for these surfaces.

The traditional method of characterising the Leidenfrost point^13^,^16^,^17^ relies on measuring the time taken for a droplet to evaporate. However, such measurements become problematic when the droplet only remains on the surface of the block for a short length of time before falling off, during which time the volume of the drop only changes by a small amount. Furthermore, there are additional complications associated with the dynamics of the system being qualitatively different from a simple droplet on a flat surface. Here, we use another feature of boiling, which is the sound produced, in order to gain insights into the boiling of droplets on ratchets.

**Figure 2:**
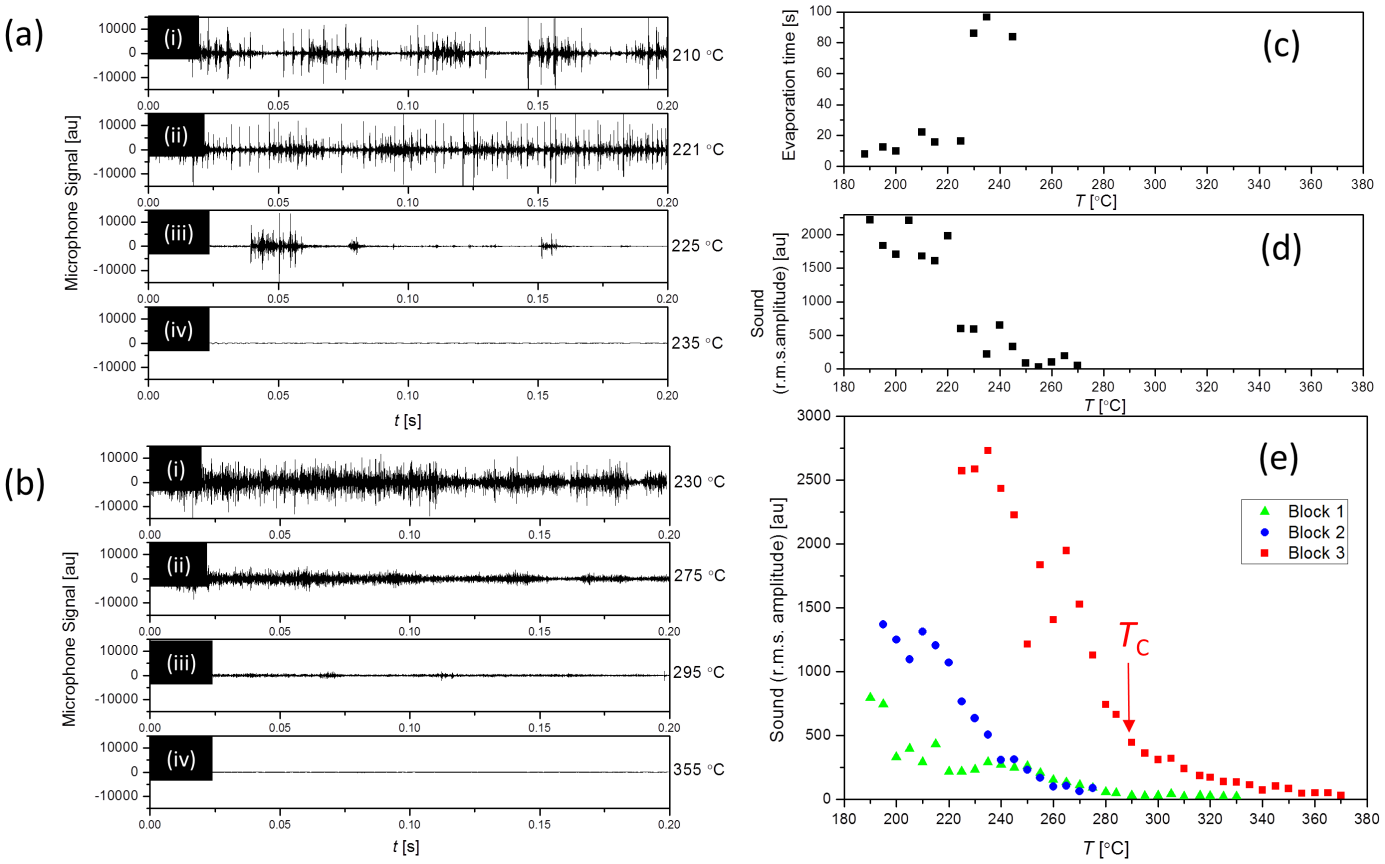
Sound of boiling droplets. (a) Sound of a droplet in a flat bowl at (i) 210°C, (ii) 221°C, (iii) 225°C and (iv) 235°C. (b) Sound of a droplet on a sharp ratchet (block 3) at (i) 230°C, (ii) 275°C, (iii) 295°C and (iv) 355°C. (c) Lifetime of a droplet in the flat bowl. (d) Root-mean-square (rms) of the microphone signal from a flat bowl. (e) rms of the sound from droplets boiling on blocks 1, 2 and 3.

As a test of this method, the sound produced by droplets boiling in a flat bowl machined into the same brass material [Figure 1(e)] was recorded at various temperatures [Figures 2(a) and (d)] (more details are provided in the Methods section). At 210°C, the droplet boils violently. Nucleate boiling towards the middle of the droplet creates vibrations and large movement, throwing liquid onto the surface that can sizzle. Long period vibrations can lead to a periodicity in the amplitude of the sound produced as can be seen in Fig. 2(a)(i). These oscillations are rather random and are absent in many measurements [such as Fig. 2(a)(ii)] and leads to a large scatter in the sound produced which we quantify by calculating the root-mean-square of the microphone signal amplitude [Figure 2(d)]. The time taken for the droplet to evaporate was also recorded [Figure 2 (c)] showing a sudden increase between T = 225°C and 230°C signifying the onset of film boiling and the Leidenfrost regime.

At 225°C, the data [Figure 2(a)(iii)] shows intervals where the droplet is close to silent, with intermittent outbursts of sound. When the droplet is close to silent, the droplet is levitating above the surface, while sound is recorded when the droplet makes contact with the hot surface. This is rather random, and occasional sizzles dominate the r.m.s. amplitude in this regime. At higher temperature still, these occasional sizzles become rare and the system becomes silent, showing that the system is fully in the Leidenfrost regime. A definition of the Leidenfrost point based on this phenomenology would be somewhat arbitrary, as there is a range of temperature over which the droplet switches between two types of behaviour. However, there is a narrow temperature window between 225°C and 230°C over which both the evaporation time (which relates to the heat transfer rate) and the average sound amplitude changes dramatically.

The sound produced by droplets boiling on the ratchets evolves in a qualitatively different manner with temperature. Figure 2(b) shows the sound produced by droplets on Block 3. At low temperature, the droplet boils noisily. As the temperature is increased, in stark contrast to droplets on a flat surface which intermittently switch in and out of film boiling, the amplitude of the sound produced decreases gradually with temperature, until it becomes silent at the highest temperatures. Towards the high temperature tail of the data [Figure 2 (e)], the scatter in the r.m.s. amplitude becomes rather small, showing the behaviour to be steady and controlled.

We hypothesize that the r.m.s. amplitude of the sound reflects the area of contact the droplets have with the brass, as we expect the contact area to determine the rate of nucleate boiling. In the entire range of temperature over which the droplets are well defined, the contact area with the ratchet is a small fraction of the total area of the droplet. At the low temperature end of this range, the ratchet teeth cut into the droplets from below, leading to a relatively large area of direct contact, and hence rapid heat transfer, nucleate boiling and in turn, a lot of sound. As the temperature increases, the droplets are forced higher, causing a reduction in the contact area. Both the sound amplitude and *θ*_C_ data shows a change in behaviour at around 290°C (marked *T*_C_ in Figs. 1(f) and 2(e)). At temperatures above *T*_C_, the teeth merely touch the bottom of the droplets, and the amount of touching is reduced when the temperature is further increased. At the highest temperatures, the droplets no longer touch the teeth due to there being sufficient film boiling to completely levitate them, and the system becomes silent.

The ability to control the height of the droplets and the way in which the droplets interact with the ratchets suggest that the droplet motion can be further controlled by embedding structure in the ratchets at different size-scales. We now demonstrate a ratchet on which the lateral direction of motion of the droplets can be controlled by changing the temperature.

### Substructure and directional control

**Figure 3:**
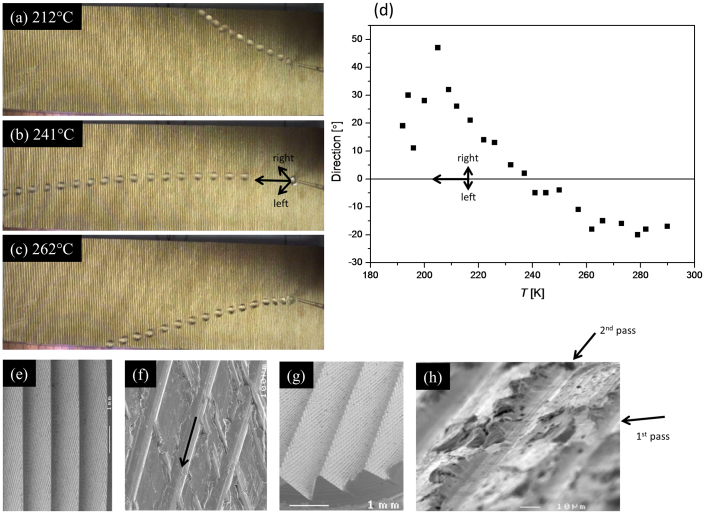
Direction control. (a), (b) and (c) show composite photographs (frames from video footage are superimposed) displaying the droplet trajectory at (a) 212°C, (b) 241°C and (c) 262°C for Block 2. (d) The final direction of the droplet as it comes off the block as a function of block temperature. (e) to (h): scanning electron micrographs of the ratchet. (e) and (f) are rotated to have the same orientation as the photographs in (a) to (c). (g) and (h) are taken from different angles.

Figure 3(a), (b) and (c) show composite photographs of the trajectories of droplets on Block 2 at three different temperatures. At low temperature, the droplets move to the right while at higher temperature, they move to the left [Figure 3(d)] with respect to the direction expected from considering the primary structure alone [arrows in Fig. 3(b)]. The paths taken by the droplets are highly reproducible. We believe that this temperature dependent directionality is caused by the substructure on each of the ratchet teeth.

Figures 3(e) to (h) show scanning electron micrographs of the ratchet taken with different magnification and orientations. Close to the step edges, there are two sets of diagonal grooves formed during the milling process. Since the teeth were milled using rotating blades [see Methods section for more information], loci described by protruding imperfections on them are inscribed onto the ratchet teeth. Scratch marks describe arcs on the teeth's sloping surfaces, crossing over to leave diamond patterns close to the steps [Figure 3 (f)] where the 2^nd^ passage of the blade leaves the more prominent grooves. i.e. near the top of the teeth, there are two sets of diagonal grooves, one scratched out after the other [Figure 3 (h)].

At the highest temperatures, the droplets move to the left. Here, the dynamics of the motion is presumably dominated by the flow of gas resulting from the film boiling and its interaction with the structures, as is the case for simpler ratchet structures deep in the Leidenfrost regime^1^,^3^,^4^,^5^. The gas flow must then be affected in such a way that the net force on the droplet has a component to the left (downwards in figs (a) to (c)). The deeper set of diagonal grooves, directed deeper into the block away from the droplet [arrow in Figure 3(f)] may preferentially guide gas flow to the left. This in turn could lead to a force on the droplet in the same direction by viscous drag^5^.

At low temperature (for example, at 212°C as shown in Figure 3(a)), the droplets make contact with the ratchet surface with a net effect of pushing the droplets to the right. The contact is confirmed by the acoustic data [Fig. 2 (e)]. At *T* = 212°C, the temperature is well below the Leidenfrost limit. Here, the dynamics of the droplet can be expected to be strongly affected by the contact and the boiling. At around 240°C where we observe the cross-over in direction, we also observe a novel dip in *θ*_C_ [Figure 1(f)], the maximum incline the droplets can climb at a given temperature, also signifying a non-trivial change in the underlying physics.

## Discussion

When the droplet makes contact with the ratchet surface, the possible mechanisms which determine the resulting net motion become complicated. It is no longer possible to neglect effects of surface tension and the forces related to the actual wetting contact with the surface. Furthermore, as demonstrated by the loud noise associated with the boiling, there must also be strong and powerful nucleate boiling and vibrations which lead to foreseeable difficulties in modelling this effect. Much remains for further investigation and we can only speculate upon possible explanations for our observations.

For block 3, for which *θ*_C_ displays a dramatic spike, the contact with the surface may enable the droplets to grip the surface by virtue of wetting, while the propulsion may be maintained by the same gas flow as in the Leidenfrost regime. The droplets make contact with the saw-teeth peaks and are suspended in between, enabling film boiling in these suspended sections. It might be expected that the grip associated with wetting is too strong to be overcome by the gas flow from film boiling. However, the vibrations caused by nucleate boiling may provide sufficient activation energy to overcome this and keep the droplets mobile. Indeed, we observe the droplets to have an opacity to the naked eye when the ratchet surface temperature is low, and photographs display more structure on the droplets indicating a violent movement of the droplet surface. At higher temperature, the droplets become smoother and more transparent. It is also likely that there is a strong influence of the greater net gas flow at lower temperature associated with greater heat transfer to the droplet, also contributing to the droplets' ability to climb steeper inclines.

As for the directional control we observe for Block 2, it is difficult to identify whether or not the smaller substructure created on the 1^st^ pass [Fig. 3(h)] has any significant effect on the droplet motion. Droplet transport to the right is likely due to a complex interplay between wetting, vibrations and gas flow and calls for further experiments.

In summary, while the detailed mechanism remains unclear, our data clearly demonstrate that additional control over the droplets can be gained by inducing more contact between the surface and the droplets. This has the effect of increasing the Leidenfrost point and offers a new handle with which to control the droplet dynamics, as well as the boiling transition itself.

## Methods

Ratchets were prepared by milling the surface of brass blocks with bits consisting of rotating blades that were scanned across the block surface. **Block 1** was milled with a bit with active blades with right-angular corners. The sloping part of the saw-tooth was inclined at 10 degrees to the horizontal while the suddenly dropping steps were inclined at 10 degrees from the vertical. For **Block 2**, a bit with blades with 60° corners was used, leading to vertically dropping steps and slopes at 30° to the horizontal. **Block 3** was patterned with a blade with a 30° corner and was positioned symmetrically with respect to the block surface. The asymmetry arises due to the malleability of the side already inscribed with the groove.

The blocks were typically 5 cm wide and 20 cm long. The blocks each had three holes drilled from the side for measuring the temperature, which was controlled by placing the blocks on a hotplate. The temperature inside the blocks was measured using thermocouples inserted into the holes. The temperatures closest to the ends were typically 5°C cooler than the centre, suggesting that the temperature of the ratchets varied by about the same amount. The entire setup was clamped onto a tilting surface fitted with a protractor, whose parallelism to the horizontal was calibrated using a spirit level. Droplets of distilled water were dropped from a pipette held a few centimetres above the ratchet, whose size was controlled by the size of the pipette opening. In this paper, we only use droplets with a spherical diameter of 3.6 mm. The motion of the droplets was recorded using a video camera enabling the position and time for each droplet to be extracted frame by frame. For sound measurements, a microphone was positioned 16 cm above the centre of the blocks. The blocks were held horizontal for all sound measurements.

## Author Contributions

RS and AG designed and conducted the experiments contributing equally. KT supervised the project. All authors discussed the results and contributed to writing the manuscript.
